# Creation and Evaluation of an Endodontic Diagnosis Training Software

**DOI:** 10.1155/2020/8123248

**Published:** 2020-07-31

**Authors:** Ebtissam M. Al-Madi, Layla Al-Bahrani, Rafif Al-Shenaiber, Samar A. Al-Saleh, Mohammad I. Al-Obaida

**Affiliations:** ^1^Department of Restorative Dental Sciences, King Saud University, P.O. Box 68004, Riyadh 11527, Saudi Arabia; ^2^Dental Department, Ministry of Health, Riyadh, Saudi Arabia; ^3^Dental Department, Prince Sattam University, Al-Kharj, Saudi Arabia; ^4^Department of Prosthetic Dental Sciences, College of Dentistry, King Saud University, P.O. Box 68004, Riyadh 11527, Saudi Arabia

## Abstract

**Objective:**

The purpose of the study is to evaluate a user-friendly, comprehensive, fully integrated web- and mobile-based application that was specifically developed to guide learners and help them practice and train in pulpal and periapical diagnosis.

**Methods:**

The software was designed for assistance in the diagnosis of the pulpal and the periapical area. The software contained questions and tests, e.g., presence or absence of signs and symptoms, cold test, percussion, palpation, and radiographic examination that the user must answer to arrive at the final diagnosis. An electronic survey was prepared to evaluate the effectiveness, productivity, and accurateness of the software. The software and the electronic evaluation survey were sent by e-mail to dental students, endodontist, general dentists, and dental interns who study or work in four Saudi dental colleges. Data was analyzed using descriptive statistics.

**Result:**

A total of 203 questionnaires were completed. Results showed that 29% of the participants were highly satisfied with the software; 40% gave a very good rating about the application satisfaction, while only 2% reported a poor degree of satisfaction with the software. Results also showed that students accurately selected the correct diagnosis but received relatively low diagnostic proficiency scores because they did not request diagnostic data in a pattern similar to experts.

**Conclusion:**

In conclusion, the software is promising as an effective and efficient tool for teaching and assessing the diagnostic skills of learners.

## 1. Introduction

The importance of advanced diagnostic skills in the practice of endodontics has been underscored in the 2008 AAE-sponsored symposium on endodontic diagnosis [1]. The diagnostic process is not a pure science, and the necessary examining equipment may not be the diagnostic tool or instrument, but the diagnostician who will perform the test and arrive at a reliable conclusion. Practitioners should be equipped with the basic requirements and skills necessary to perform this procedure, including knowledge, training, interest, curiosity, patience, the art of listening, and above all common sense.

Endodontic diagnosis is similar to a puzzle, in that diagnosis cannot be made from a single, isolated piece of information [2]. The clinician must systematically gather all of the necessary information to make a “probable” diagnosis. When taking the medical and dental history, the clinician should already be formulating in his or her mind a preliminary, but logical, diagnosis especially if there is a chief complaint. The clinical and radiographic examinations, in combination with a thorough periodontal evaluation and clinical testing (e.g., pulp and periapical tests), are then used to confirm the preliminary diagnosis.

Diagnostic skills need practice, training, and experience to develop. Educators and instructors have the responsibility of providing as many experiential opportunities to allow learners (whether undergraduate, postgraduate students, or clinicians taking a CE course) as necessary for them to gain confidence. However, the limited number and variety of clinical experiences a student is exposed to during their education makes it difficult to gain efficiency and expertise. Dental students in most dental schools have a few years of supervised clinical patient treatment, which may not be sufficient to see enough cases to the level of proficiency. As for other learners (practitioner), an expert consultation might not be at hand.

Computer-Assisted Learning (CAL) or Computer-Assisted Instruction (CAI) has become a popular method to provide information to students, patients, and practitioners. [3]. CAL has many advantages as learners benefit by individualizing the educational process, allowing them to work at their pace and to repeat the learning program as much as required [4, 5]. The primary goal of educational software is to teach and/or assess students. Simulation programs, in the form of Computer-Assisted Learning (CAL), can provide dental students that have a limited opportunity to practice diagnosing patient problems with additional experience. These products include tutorials, hypermedia, drill and practice applications, simulations, games, and assessments [6]. Multimedia programs have been developed for training dental students and dental practitioners in decision-making and problem-solving in endodontics, such as the SimEndo I [7]. This showed a positive affective response to the experience, with a high satisfaction by students. Case simulations could help teach endodontic diagnosis through feedback and the opportunity for repetition and correction of errors. CAL methods can be used to aid traditional methods to develop overall knowledge, prepare the student and practitioner for real-life situations, and help expose the learner to a variety of different cases, rather than be limited to individual experiences [8]. A software would act as guidance for learners (student as well as practitioner) to help them reach the final diagnosis. Mobile applications are readily available and can be used to assist students in a more convenient manner.

The purpose of the study is to evaluate a user-friendly, comprehensive, fully integrated web- and mobile-based application that was specifically developed to guide learners and help them practice and train in pulpal and periapical diagnosis.

## 2. Materials and Methods

An English language web-based mobile endodontic and periapical diagnosis training application was developed using JQuery Mobile Web framework and JavaScript technology platforms. The mobile iOS application was developed to serve as an adjunct to the process of learning. The application could be used as a decision support aid using real case scenarios, or a training tool with multiple case scenarios imbedded. The requested data fields included the affected tooth number (based on FDI World Dental Federation numbering system), extraoral and intraoral clinical examination questions, presence or absence of signs and symptoms, cold test results, percussion, palpation, periodontal probing, and radiographic examination. A decision tree with specific treatment options was structured; then definite outcomes and probabilities to occur were determined. Finally, the most preferable treatment was estimated by computed calculations. The software also suggested treatment options and predicted prognosis. There was an option to upload radiographs either directly from the phone camera or scanned and uploaded to the application. Each user could log in using his/her own username and password to the system then upload patient personal data, affected teeth, and signs and symptoms that were collected from the patient; then the program gave a diagnosis and suggested treatment options for the case according to the information analyzed by the program. This application was developed to work with numerous decisions related to patient's signs and symptoms included in the design.

The researchers visited 4 dental schools to introduce the application (King Saud University-College of Dentistry (KSUCD), Riyadh Al-Elm University-College of Dentistry (REUCD), Imam Abdulrahman University-College of Dentistry (IAUCD), and King Abdulaziz University-College of Dentistry (KAUCD) at the beginning of the school year through a short presentation. All dental students, interns, general dentists, endodontic board residents, and endodontists were asked to participate in the study. Names and emails of all interested participants were collected during the introduction, and the application was sent by e-mail to 408 potential participants with a link to download the software and instructions explaining its benefits and usage.

A feedback survey was developed that consisted of 20 close-ended questions, and 3 open-ended questions were included in the application (Table 1). A pretest for the survey was evaluated by two endodontists for content validity, internal consistency, and interrater reliability. The pretest was also evaluated by 10 participants, representative of the sample, to verify clarity and appropriateness of the survey questions. The survey was resent to the pilot group a second time 2 weeks later to test reliability. The statements questioned the ease of learning to use the application, visual appeal, organization of information, ease of use, reliability, appropriateness of application for different levels, satisfaction, effectiveness, sequence of data entry speed, comprehensiveness of list of signs and symptoms provided, effect on productivity, capability, clarity of icons, accurateness of the presented diagnosis, comparison of application to traditional systems to assist diagnosis, and probability of using of the application in the future. A follow-up e-mail was sent to participants to encourage them to respond after 1 month of use.

A 5-point Likert scale with 5 = excellent and 1 = poor was used. Data were entered and analyzed using SPSS PC+ 16.0 version statistical package. Descriptive statistics were analyzed.

## 3. Results

A total of 203 questionnaires were completed (111 females, 92 males) resulting in a 50% response rate. Distribution according to levels is shown in Table 2.

Most of the participants (43%) found that it is easy to learn how to use the application, whereas only 1% found it difficult. The visual appeal was reported as excellent by 41% of the participants. However, only 42% found the organization of the information and terms used in the application very good. More than half (54%) of the participants reported that it is easy to use the application. Approximately 43% of the participants thought that applications such as this one was reliable, whereas only 1% found it was not. Regarding effectiveness, 41% found the application very effective, whereas only 3% did not. Out of the sample participants, 40% said the application had a very good effect on their productivity after use. More than half (64%) of the participants stated that the diagnosis provided was accurate, when used in case scenario mode. However, in comparison to traditional (paper-based) systems used to assist in the diagnosis procedure, 90% reported that the use of the application is better. Two-thirds (68.5%) of the participants confirmed that there is a high or very good chance to use the software in the future, while only 7% reported that there is a poor chance of using the software in the clinic.

**Figure 1:**
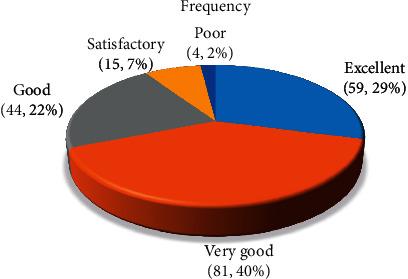
Satisfaction rates of the software.

Overall, 29% of the participant were highly satisfied with the software; 40% gave a very good rating about the application satisfaction, while 2% reported a poor degree of satisfaction with the software. Satisfaction rate of the software is shown in Figure 1. No significant difference was found among level of education between respondents, college, nor among GPA of students.

## 4. Discussion

Endodontics practice requires knowledge and problem-solving skills in order to derive an accurate diagnosis, treatment plan, and patient prognosis [7]. Treatment decisions are the result of complex cognitive process [9].

It is a personal and cognitive experience, and many of the qualities of a good diagnostician are of an interpersonal nature and are based on interest, intuition, curiosity, and patience. Other skills the diagnostician must master include knowledge and experience. Knowledge is the most important asset the dentist must possess via complete understanding of basic information regarding diagnosis of pulpal pathosis, ability to formulate the right treatment plan for each case, and evaluating the given treatment and expected prognosis. Diagnosis can be difficult, and it is necessary to obtain a differential diagnostic approach. The decision to undertake root canal therapy should not be made in isolation. Patient considerations including medical conditions, physical impairment, and motivation to maintain oral health must be taken into account [10].

Historically, there have been a variety of diagnostic classification systems advocated for determining endodontic disease. In 2008, the American Association of Endodontists [11] standardized diagnostic terms used in endodontics [12]. The goals were to propose universal recommendations regarding endodontic diagnoses and develop standardized definitions of key diagnostic terms that will be generally accepted by endodontists, educators, test construction experts, third parties, generalists, specialists, and students [13, 14].

The present study described a mobile web software application that has been created to be used for endodontic diagnosis training. Benefactors can include dental students, interns, and general dentists who need help with additional practice in diagnosing patient endodontic problems. The simulated program assesses diagnostic proficiency and accuracy (through choice of correct answers) by comparing it to an expert diagnosis. The software guides the learner through the sequence of patient data requests to arrive at a diagnosis. It is important to evaluate software developed for this kind of training to make sure participants are likely to use it. It must be easy to learn and easy to use, visually appealing, and organized in a clear way. Even though working with e-learning has been shown to lead to better decision-making skills [15, 16], participation in e-learning modules differs greatly when certain user acceptance aspects are not observed when designing the program [17]. In the case of our software, this was reported between 41 and 54%, which indicates that there is a room for improvement. This software shows promise as an effective and efficient tool for teaching and assessing diagnostic skills of undergraduate students. However, it was observed that students did not request diagnostic data in a pattern similar to experts, which could reduce the reliability and accuracy of diagnosis, which might give faculty and teachers an indication of where lack of knowledge lies, and can help teachers in pinpointed causes of misdiagnoses among learners. Overall, participants rated it as very good or excellent (40% and 29%), and they found it useful as a source of information and felt that it helped their learning experience. Most of the participants had positive reviews regarding the software. They thought it gave them a good exercise and independent practice in diagnosis. This might be primarily due to the repetitive training opportunity that gave them more confidence as they used the application. In addition, independent, interactive e-learning modules have been shown to be successful in the delivery of foundational knowledge, as the interactive content positively engaged new generation dental students. Interactive modules have been recognized as a valuable learning resource and were preferred over the classroom, but not necessarily seen as a total replacement for a traditional course. Thoughtful integration of e-learning into the curriculum is required and may be best if combined with some classroom activities or seminars to address the students' desire for faculty interaction [18]. On the other hand, there were a small number of participants who were not confident using the software in real cases. They felt the software might not give them accurate diagnosis. It is important to emphasize that this is a training tool and not a replacement for clinical judgment. Decision support tools are simply adjunctive methods to either confirm or dispute the decision to assist the clinician or to guide and train students.

There is some evidence to suggest that CAL is time efficient compared to traditional methods [19]. Endodontic diagnosis is a part of everyday dental practice that needs time and special (scientific and artistic) skills from the practitioner. To be an effective treatment provider, the practitioner should be exposed to many case scenarios to adequately practice decision-making. Using CAL will give them a chance to do so in a safe environment, repeatedly, in order to gain competency and confidence. Part of the success of CBL features comes from empowering learners to learn at their own pace, providing them with richer interactions with learning materials, which were not otherwise feasible [20]. The integration of case-based e-learning modules in a learning environment might provide a successful additional learning opportunity for learners to acquire necessary clinical decision-making skills for responsible patient care [21].

There are many benefits to extending the use of such software. Information can be easily uploaded and programmed to serve as case database and can be continuously updated and shared to be used by all. Engaging students in the creation of content can be a good way to help faculty cope with the increasing demand for learning material [22]. It is relatively easy, accessible, and user friendly. It does not occupy much space in phone memory. It can facilitate communication between dentists for consultation via teledentistry. It has been used in oral medicine [23] and oral and maxillofacial surgery [24] and as a screening tool for oral diseases [25]. To increase participation, the next steps could be to apply this e-learning application as a mandatory training during endodontic courses and to provide it in line with the objectives for any exams [26].

Despite the limitations of the study, evaluation of the e-learning platform as provided in the application developed for this study was positive and seemed helpful in motivating learners practice clinical decision-making in self-directed manner.

## Figures and Tables

**Table 1:** Survey questions.

Question	Answer
(1) Learning to use the application	(1) Excellent (2) Very good (3) Good (4) Satisfactory (5) Poor
(2) Visual appeal of application	
(3) Organization of the information	
(4) Terms of use in the application	
(5) Ease of use	
(6) Reliability of the application	
(7) Appropriateness of application for different levels	
(8) Application meets expectations	
(9) Satisfaction of using the application	
(10) Effectiveness of the application	
(11) Effect of the application on speed in diagnosis	
(12) Effect of software on productivity	
(13) Capability of the application	
(14) The chance of using of the application in the future	
(15) Clarity of icons that are used to assist navigation (e.g., back, exit, and save)	
(16) Sequence of data entry	
(17) Comprehensiveness of list of signs and symptoms provided	
(18) Accurateness of the given diagnosis	
(19) Comparison of application to traditional systems to assist diagnosis	
(20) Rating the software from 100%	

(21) Please add your comments regarding benefits of creating similar applications?	Open-ended questions
(22) Please indicate any drawbacks that faced you while using this application?	
(23) Please give us any suggestions that can be made for update	

**Table 2:** Level of students participated in the research.

	Frequency	Valid percent
3rd	28	13.8
4th	21	10.3
5th	26	12.8
6th	29	14.3
Intern	71	35.0
GP	13	6.4
Endodontist	5	2.5
Others	10	4.9

Total	203	100.0

## Data Availability

The data used in this study are available from the corresponding author upon request.
